# Opinion seeking behaviour of healthcare providers in ambulatory cardiovascular care in Germany: a cross-sectional study

**DOI:** 10.1186/s12913-022-08667-y

**Published:** 2022-11-23

**Authors:** Patrick Hennrich, Christine Arnold, Pia Traulsen, Frank Peters-Klimm, Michel Wensing

**Affiliations:** Heidelberg University Hospital, Im Neuenheimer Feld 130.3, 69120 Heidelberg, Germany

**Keywords:** Opinion leader, Opinion seeking, Coordination, Cardiology, Ambulatory, Germany, General practitioners, Cardiologists, Medical specialists

## Abstract

**Background:**

Healthcare providers’ inclination to seek or lead other providers’ opinions on clinical topics may influence healthcare practices, particularly regarding their alignment across different providers in controversial domains. This study aimed to explore opinion-seeking behaviours of general practitioners and their impacts on clinical opinions in ambulatory cardiovascular care in Germany.

**Methods:**

Between 2019 and 2021, we performed a written survey in two samples of general practitioners and one sample of self-employed cardiologists in three German states. The general practitioners were asked to identify a person they deemed influential on their views on cardiovascular conditions. Their self-perceived opinion leadership and opinion seeking behaviours were then measured, using a validated 12-item-questionnaire. General practitioners and cardiologists were requested to indicate their agreement with three potentially controversial aspects of cardiovascular ambulatory care. Potential impacts on the general practitioners’ views, including local cardiologists’ opinions, were examined using multi-level linear regression models.

**Results:**

A total of 129 general practitioners and 113 cardiologists returned the questionnaire. 68.50% of general practitioners named an opinion leader, mainly cardiologists outside of their practice. General practitioners perceived themselves as opinion seeking and as opinion leading at the same time. Views on the presented controversial topics were mixed among both general practitioners and cardiologists. Self-reported opinion leadership behaviour of general practitioners was associated with their views on one of the three topics. No such associations were found for opinion seeking behaviours and the views of local cardiologists.

**Conclusion:**

While most general practitioners named a cardiovascular opinion leader and saw themselves as opinion seeking regarding cardiovascular issues, they simultaneously perceived themselves as opinion leading, suggesting that opinion leadership and opinion seeking are not mutually exclusive concepts. The views of local cardiologists were not associated with the general practitioners’ view, suggesting that local medical specialists do not necessarily influence the surrounding opinion seekers’ views per se.

**Trial registration:**

We registered the study prospectively on 7 November 2019 at the German Clinical Trials Register (DRKS, www.drks.de) under ID no. DRKS00019219.

**Supplementary Information:**

The online version contains supplementary material available at 10.1186/s12913-022-08667-y.

## Background

The role of the social environment in the development of individual opinions is well-established, also in research on physicians and other healthcare providers [1]. Opinions of colleagues or peers are an important point of reference, which complement and may override other sources, such as professional training, clinical experience, and practice guidelines. Clinical opinion leaders have been identified in a range of settings, showing that individuals in this role rotate within a few years of time [2]. The involvement of clinical opinion leaders has been used to harness continuing professional education and other initiatives to implement evidence-based medicine and to improve clinical practice [3]. On the other hand, opinion-seeking behaviours of healthcare providers have been less well studied, which may reflect the assumption that opinion seeking is simply the opposite of opinion-leading.

In the German healthcare system, most patients who suffer from cardiovascular diseases receive medical care from a general practitioner (GP). In Germany, this is a primary care physician with vocational training in general practice or a medical specialist in internal medicine or paediatrics. Patients attend a cardiologist when new (complex) conditions emerge, in urgent cases that demand specialist cardiology knowledge, and possibly for up to half-yearly appointments. Primary care cardiologists are mainly self-employed and based in their own practices while hospitals are largely restricted to inpatient care. This structure is similar to some countries, while in other countries both ambulatory and inpatient cardiology care are provided in hospitals [4, 5].

Given the fragmented structure of healthcare, coordination of cardiovascular care can be challenging in the German context. In addition, physicians treating cardiovascular conditions have multiple points of reference, including clinical practice guidelines, continuing medical education activities (e.g. conferences) and integrated care programmes (initiated by health insurers) [6]. Cardiovascular guidelines that aim to support both GPs and medical specialists are the National Care Guidelines (“Nationale VersorgungsLeitlinien”) for heart failure and coronary heart disease [7, 8]. In addition, four cardiovascular guidelines from the German College of General Practitioners and Family Physicians (DEGAM) cover stroke, chest pain, prevention-related counselling and anticoagulation medication [9]. Numerous other cardiovascular guidelines are primarily targeted at cardiologists and other medical specialists, such as those of the German Cardiac Society, which often are slightly adapted versions of guidelines published by the European Society of Cardiology [10].

This raises the question whether GPs tend to resort to opinion leaders as a point of reference for their professional views on the clinical management of cardiovascular conditions. On the one hand, e.g., GPs could resort to orient themselves on local cardiologists and cardiology guidelines. This would ensure decisions that are in line with the respective medical specialist’s view. Given the traditional reputational hierarchy in medicine, which may be particularly strong in Germany, one might expect defined pathways where GPs are geared to medical specialists anyway when it comes to cardiovascular conditions. Ultimately, this would imply that GPs primarily see themselves as opinion seekers regarding cardiovascular conditions and seek opinions from cardiologists accordingly.

On the other hand, GPs gather medical knowledge and clinical experience in primary care, partly through other GPs who are role models, which may result in views that differ from specialist cardiology. Research confirmed that GPs’ decision making and ways of arriving at a diagnosis differ from those of medical specialists in an ambulatory or inpatient setting [11–14]. The underlying reason may be that the patient population in general practice tends to have a lower a priori risk of severe disease and unfavourable outcomes. Furthermore, GPs might have a more holistic image of the patient since they regularly are familiar with the patient’s other health problems and living situation. Self-confident GPs see themselves as experts on the specific patient and hence might perceive themselves to be a point of reference for others regarding that patient. This would imply that these GPs tend to see themselves as opinion leaders instead of seeking advice from others. When it comes to cardiovascular issues, they therefore may not have a clear preference regarding other opinion leaders, since they are mostly driven by what they deem most suitable for their patient who they know in-depth.

Regarding the conceptualisation of opinion leadership, we followed the definition of Rogers [15] who described opinion leadership as: “[…] the degree to which an individual is able informally to influence other individual’s attitudes or overt behaviour in a desired way with relative frequency. Opinion leaders are individuals who lead in influencing other’s opinions” [15]. Hence, the individual’s influence on others’ opinions is not (necessarily) based on their formal position, e.g. in an organization or other kind of hierarchy, but also on informal assets regarding competence, accessibility and normative conformity [3].

Our study aimed at answering three interconnected questions: (1) Who are cardiovascular opinion leaders for GPs? (2) To what extent do GPs perceive themselves as cardiovascular opinion leaders and opinion seekers, and which factors are associated with these perceptions? (3) To what extent are GPs’ self-perceptions of opinion-seeking and opinion-leading behaviours related to their views on specific aspects of cardiovascular care?

## Materials & methods

### Study design

A written survey was conducted in a sample of ambulatory care providers (general practitioners and cardiologists) in Germany. The study was part of the larger ExKoCare research project, conducted between 2019 and 2022, which aimed at exploring mechanisms that potentially influence coordination and uptake of recommended cardiovascular care in an ambulatory setting using a mixed-methods approach [16]. The study was approved by the Ethics Committee of the medical faculty of Heidelberg (“Ethikkommission der Medizinischen Fakultät Heidelberg”) under ID S-726/2018. Written informed consent was obtained from each participant where required (see “Study population and sampling” for details). Structured written surveys were mailed out to GPs and cardiologists in the German states of Baden-Wuerttemberg (approximately 11 million inhabitants in 44 counties), Rhineland-Palatinate (approximately 4 million inhabitants in 36 counties) and Saarland (approximately 1 million inhabitants in 6 counties).

### Study population and sampling

The study focused on GPs and cardiologists in ambulatory care. A total of three samples was planned with regard to the underlying ExKoCare project that included several topics.

In the first sample, we aimed to recruit a total of 40 general practices for the full ExKoCare project. Eligible practices in each state were identified through the publicly available online database of the respective association of statutory health insurance physicians. From previous experience with the recruitment of general practices [17], we initially expected a participation rate below 5%. Hence, we eventually approached a total of 1842 practices in 25 counties. Sampling was performed with regard to county size and structure, so that both urban and rural areas were included. Practices were approached via fax (which is one of the most widespread ways of communication for physicians in Germany). They were presented a short summary of the study and were asked to indicate their interest. Each practice that was interested in the study received a letter containing the necessary background information as well as an informed consent form. Written informed consent to participate in the project was required from each practice owner and from each physician within a general practice. Whenever written informed consent could not be obtained, the respective person or practice was not included in the study. After 3 months, practices that initially indicated their interest in the study but did not respond to our letter received a reminder via fax where they were asked to indicate whether they were still interested or not and whether they still had the documents we sent out to them or not. Eventually, a total of 42 practices (2.3%) agreed to participate in the project. Within these practices, we identified and approached a total of 56 GPs for the present study. Since the first sample was planned to take part in the ExKoCare project as a whole (over the course of 2 years) and not only the study presented here, practices were offered a monetary incentive of 250 Euros for completing the project.

In the second sample, we approached a total of 681 additional general practitioners in 21 counties in the three federal states through an anonymous, written questionnaire accompanied by necessary background information. The physicians were approached via letter, which contained a short cover letter, necessary background information and the questionnaire itself. Sending back the completed questionnaire was interpreted as informed consent. Since these GPs were not involved any further in the ExKoCare project and only had to complete an anonymous questionnaire, an additional declaration of consent was waived by the Ethics Committee of the medical faculty of Heidelberg. No additional incentives were offered to the physicians in the second sample.

Finally, in the third sample, a full census of cardiologists was approached for participation. We identified a total 534 self-employed cardiologists in the three states, all of whom we invited for participation. Similar to the second sample, the Ethics Committee of the medical faculty of Heidelberg waived the written declaration of consent due to the study being anonymous and the cardiologists not being involved any further in the ExKoCare project. Therefore, sending back the completed questionnaire was interpreted as informed consent here as well. The physicians were also approached via letter, which contained a short cover letter, necessary background information and the questionnaire itself. No additional incentives were offered to the physicians in the third sample.

### Measurements and data-collection

Three different questionnaires were distributed in the ExKoCare project. Since the study presented here was only one part of the project, the questionnaires also covered topics that are not part of this research paper: (1) Each GP in the first sample received a questionnaire covering sociodemographic data, information exchange within and outside of the practice, team climate, cardiovascular issues, influences on patient care, perceived opinion leadership and opinion seeking as well as views on cardiovascular recommendations. In total, the questionnaire contained 49 questions. (2) The 681 additional GPs in the second sample described above received a more condensed, anonymous questionnaire covering sociodemographic data, information exchange outside of the practice, influences on patient care, perceived opinion leadership and opinion seeking as well as views on cardiovascular recommendations. In total, the questionnaire contained 32 questions. (3) The 534 cardiologists in the third sample received a condensed, anonymous questionnaire as well, covering sociodemographic data, information exchange outside of the practice and views on cardiovascular recommendations. In total, the questionnaire contained 15 questions.

To examine whether or not GPs could point out a (medical) opinion leader for cardiovascular conditions in their surroundings, we asked them to indicate if there was someone asserting influence on their views through the question: “Is there currently a professional who affects your opinion on cardiovascular conditions?”. Respondents could choose between another physician within their practice, a GP outside of their practice, a cardiologist outside of their practice, someone else outside of their practice or they could indicate that they cannot name such a person. This approach was derived from the sociometric method that identifies opinion leaders by asking respondents about who they would turn to when looking for advice [15]. The focus of measurement here therefore was the personal influence of certain individuals as perceived by others – unlike in alternative approaches, where opinion leadership is measured e.g. by the degree of individual involvement in communication only [18].

To measure GPs’ self-perception regarding opinion seeking and leading, we translated a validated questionnaire by Flynn et al. [19]. The questionnaire originates from marketing research, where opinion leadership has been extensively studied. According to the developers, it is applicable to “specific product or service domains” [19]. Hence, an adaptation to medicine and, more precisely, to the field of cardiology seemed justifiable to us, since the instrument specifies no topics ex ante and cardiovascular care can, in a broader sense, be seen as a service for patients. With a total of 12 items, the questionnaire is meant to identify how strongly respondents perceive themselves as opinion leaders (6 items) and as opinion seekers (6 items) in a specific domain. Each item consists of a statement on how the respondents perceive their influence on others’ opinions on a given topic and if they turn to others to get their opinion or advice on said topic. Each statement has to be rated on a scale from 1 (“totally disagree”) to 5 (“totally agree”).

Regarding views on cardiovascular recommendations, after consultation with clinical experts we included three statements in our questionnaire that relate to potentially controversial aspects of cardiovascular care. Respondents had to rate each statement on a scale from 1 (“totally disagree”) to 5 (”totally agree”) or they could indicate that they did not have an opinion on the respective statement. The statements were: (1) For patients suffering from coronary heart disease, a therapy with highly dosed statins has to be initiated. (2) The general practice always has to check the brain natriuretic peptide (BNP)-parameter when a patient suffers from dyspnoea and possibly suffers from heart failure. (3) Every patient suffering from coronary heart disease and hypertension needs to reach a systolic reading below 130 mmHg.

These statements are consistent with prevailing cardiology guidelines for cardiovascular care. The first statement originated from the 2018 American Heart Association’s Guideline on the Management of Blood Cholesterol, where, for patients with clinical atherosclerotic cardiovascular diseases, a high-intensity statin therapy is recommended [20]. The second statement was derived from the European Society of Cardiology’s guideline on heart failure as well as the German “Nationale VersorgungsLeitlinie Chronische Herzinsuffizienz” (national care guideline on heart failure). Both guidelines recommend checking BNP-parameters in patients where symptoms for heart failure are present as soon as possible during the first medical contact [7, 21]. In Germany, the first medical contact typically is the GP, unless there is an emergency. The third statement originated from a controversy between the American 2017 guideline on high blood pressure in adults where blood pressure is classified as hypertension when there is a systolic reading > 130 mmHg [22] and the conflicting statement by the European 2018 guidelines for the management of arterial hypertension where hypertension is assumed only when there is a systolic reading > 140 mmHg [23].

### Data analysis

Questionnaires returned to us were scanned, reviewed for missing values, invalid or ambiguous responses and a dataset was created.

Sociodemographic data of participants were analysed descriptively using mean values as well as relative and absolute frequencies. Data on opinion leaders identified by GPs were analysed descriptively using relative and absolute frequencies. The two scales on opinion leadership and opinion seeking were calculated by recoding negatively formulated items in the opinion leadership questionnaires and then calculating the mean values of each respondent on the 6 items for both scales. Recoding was performed in a way that high mean values indicated high opinion leadership/seeking on the final scales. Both scales had a value between 1 and 5 each, with 1 indicating a weak self-perception as opinion seeker/leader and 5 indicating a strong self-perception. We allowed up to two missing values on each scale, so each participant had to answer to at least 4 items per scale to be included. Missing values were imputed row-wise using the patient’s mean value of the 4 items that were completed. Participants with more than 2 missing values on the respective scale’s items were not included in the scale and the respective analyses. Since, as we mentioned, the scales were validated but did not originate in health research, we performed an additional reliability-analysis using Omega. We followed the recommendation of Flora (2020) to calculate categorical Omega (ω_u−cat_) for unidimensional scales consisting of 5 or less response options per item [24].

Possible determinants of self-perceived opinion leadership/seeking were analysed using linear regression with the respective scale as a dependent variable. Independent variables were age in years, sex (male/female) and whether or not the respondents could identify an opinion leader (yes/no).

Opinions on the three cardiovascular statements were analysed descriptively for GPs and cardiologists. Respondents with missing values and respondents who indicated that they had no opinion were excluded from the analysis of the respective statement. Since the data on the three statements were not normally distributed, descriptive analysis was performed using median values and interquartile ranges (IQR). Then, Wilcoxon rank sum tests were performed to compare opinions between GPs and cardiologists. Finally, three multi-level linear models were calculated for GPs to examine potential influences on their opinions regarding the statements. Covariates were: The values on the opinion leadership/seeking scales, sex (male/female), age (in years), state (Baden-Wuerttemberg yes/no), whether the GP identified an opinion leader (yes/no) and the mean rating of each statement by the cardiologists in the respective GP’s region. The latter was performed to further examine associations between the local cardiologists’ and the GPs’ opinions within each region. Regions were included as random effects. All analyses were performed using R [25] with a significance level of α = 0.05.

## Results

A total of 47 (83.93%) physicians within the 42 practices returned the questionnaire. Of the additional 681 GPs who received an anonymous questionnaire, 11 mailings were returned to sender. From the remaining 670 physicians, a total of 82 (12.24%) responded, resulting in a total of 129 GPs. Of the 534 cardiologists who received an anonymous questionnaire, a total of 113 (21.16%) responded.

### Sample description

Table 1 shows basic sociodemographic data for general practitioners and cardiologists.

**Table 1:** Sociodemographic data for general practitioners and cardiologists

	General practitioners (n = 129)	Cardiologists (n = 113)
Sex (n (%))		
male female not specified	65 (50.40) 64 (49.60) 0 (0.00)	91 (80.50) 21 (18.60) 1 (0.90)
Age in years (mean (standard deviation (SD))) [n]	58 (9.65)	55 (8.31) [111]
State (n (%))		
Baden-Wuerttemberg Rhineland-Palatinate Saarland	74 (57.40) 45 (34.90) 10 (7.75)	64 (56.60) 42 (37.20) 7 (6.19)
Established since the year (mean (SD)) [n]	2002 (11.86) [125]	2009 (8.95) [110]
Occupational title (n (%))		
Specialist in general medicine	104 (81.90)	-
GP with a specialization in internal medicine	20 (15.70)	-
Other	3 (2.40)	-
Internal medicine with a focus on cardiology	-	59 (52.20)
Internal medicine and cardiology	-	39 (34.50)
Internal medicine without a focus	-	14 (12.40)
Occupational status (n (%))		
Full-time occupation (35 h a week and more)	107 (82.90)	101 (89.40)
Part-time occupation (< 35 h a week)	20 (15.50)	12 (10.60)

### Presence of an opinion leader on cardiovascular conditions

Results on whether GPs perceived an opinion leader regarding their view on cardiovascular conditions showed that the majority of GPs (55.90%) named a cardiologist outside of their practice as a professional with impact on their own opinion on cardiovascular conditions. This was followed by respondents not being able to name such a person (31.50%). Other GPs inside one’s own practice were seldom named (5.40%), the same goes for other persons outside one’s own practice that were neither GPs nor cardiologists (7.20%). GPs outside of the practice were not named at all.

### Self—perceived opinion leadership and opinion seeking

The theoretical range of values for both opinion leadership and opinion seeking varied between 1 (low) and 5 (high). Imputation was necessary for 6 respondents on the opinion leadership scale (1 missing value in 4 cases, 2 missing values in 2 cases) and for 1 respondent (1 missing value) on the opinion seeking scale. Two respondents did not answer any of the items and were hence excluded from each scale. The internal consistency of the scales for opinion leadership and opinion seeking was satisfying with ω_u−cat_ = 0.98 (standard error (SE) = 0.01, 95%-Confidence Interval (CI) [0.98–0.995]) for the opinion leadership scale and ω_u−cat_ = 0.97 (SE = 0.05, 95%-CI [0.96–0.99]) for the opinion seeking scale.

The GPs scored a mean of 2.57 (standard deviation (SD) = 0.90, n = 127) on the opinion leadership scale and a mean of 3.71 (SD = 0.83, n = 127) on the opinion seeking scale.

Even though total scores on the opinion seeking scale were higher than on the opinion leadership scale, this did not allow for the conclusion that both scales were associated with each other: The low correlation coefficient between the opinion leadership and opinion seeking scale (r = 0.18 (r = 0.22 when corrected for attenuation)) among GPs indicated that high values on the opinion leadership scale do not necessarily go along with low values on the opinion seeking scale and vice versa. Especially illustrative examples were isolated cases where respondents saw themselves neither as opinion leaders nor as opinion seekers or, in contrast, others with high values on both scales.

Regarding the question what influences self-perceived opinion leadership, regression analyses showed an effect of the presence of other opinion leaders: People who were able to name someone who influenced their views on cardiovascular conditions scored significantly higher on the opinion leadership scale (Estimate (b) = 0.46, 95%-CI: [0.11; 0.81], p = 0.01) than those who could not name someone. Further covariates did not significantly affect self-perception. Looking at the F-statistics with F[4] = 1.99, p = 0.10 and the adjusted R² = 0.04, the model showed a poor goodness of fit with little explanatory power.

For opinion seeking, we found an effect of the presence of opinion leaders as well: People who were able to name someone influencing their view on cardiovascular conditions had significantly higher scores on the opinion seeking scale (Estimate (b) = 0.45, 95%-CI: [0.13; 0.78], p = 0.007) than those who could not name someone. Furthermore, there was an effect for sex, with men having significantly lower scores on the opinion seeking scale (Estimate (b) = -0.34, 95%-CI: [-0.67; -0.01], p = 0.04). Further covariates showed no significant influences on self-perception. In this model, the F-statistics were statistically significant with F[4] = 4.88, p = 0.001 and an adjusted R² = 0.13.

### Views on cardiovascular care and associated determinants

Physicians views on the three cardiovascular topics are presented in Table 2. Besides missing values, one GP indicated not having an opinion on statement 1 and was hence excluded from the analysis as well.

**Table 2:** Respondents’ median agreement with statements derived from cardiovascular guidelines

Statement	General practitioners (median (IQR) [n])	Cardiologists (median (IQR) [n])
For patients suffering from coronary heart disease, a therapy with highly dosed statins has to be initiated.	4 (2) [125]	4 (1) [112]
The general practice always has to check the BNP-parameter when a patient suffers from dyspnoea and possibly suffers from heart failure.	4 (3) [125]	4 (1.25) [112]
Every patient with coronary heart disease and hypertension needs to reach a systolic reading below 130 mmHg.	4 (2) [127]	3 (2) [112]

Legend: IQR = Interquartile range. Response options ranged from 1 (“Do not agree at all”) to 5 (“Fully agree”)

Overall, respondents leaned towards agreement with the statements and median responses were similar between GPs and cardiologists. However, as the IQR shows, there was also some variation. A check for statistically significant differences between GPs and cardiologists regarding their views on the statement showed that cardiologists agreed significantly stronger with statement 1 than GPs (Wilcoxon rank sum test W = 4503.5, p < 0.001). This was not the case for statement 2 (W = 7207, p = 0.69) and statement 3 (W = 7452.5, p = 0.51).

Table 3 summarizes the findings of the regression analyses, which examined factors that are possibly associated with the GPs’ views on the three selected topics.

**Table 3:** Multilevel regression of the GPs’ agreement with each statement on 7 predictors

	Statement 1: Therapy with highly dosed statins for Coronary Heart Disease (CHD)-patients.			Statement 2: BNP-check by the GP when patient suffers from dyspnoea and possibly heart failure.			Statement 3: Systolic reading below 130 mmHg for every patient with CHD and hypertension.		
***Predictors***	***Estimate (b)***	***CI***	***p***	***Estimate (b)***	***CI***	***p***	***Estimate (b)***	***CI***	***p***
Age (years)	0.04	0.01 ; 0.07	0.011*	0.03	0.00 ; 0.06	0.031*	0.03	-0.00 ; 0.06	0.075
Sex (ref. female)									
Male	-0.37	-0.94 ; 0.20	0.202	-0.52	-1.05 ; 0.01	0.053	-0.39	-0.95 ; 0.16	0.164
State (ref. not Baden-Wuerttemberg)									
Baden-Wuerttemberg	-0.26	-0.83 ; 0.31	0.370	-1.18	-1.78 ; -0.58	< 0.001***	0.51	-0.03 ; 1.05	0.063
Respondent identified an opinion leader	-0.04	-0.64 ; 0.56	0.899	-0.12	-0.68 ; 0.45	0.681	-0.29	-0.87 ; 0.30	0.333
Value on opinion leadership scale	0.27	-0.04 ; 0.59	0.089	0.30	0.01 ; 0.06	0.041*	0.14	-0.17 ; 0.44	0.373
Value on opinion seeking scale	-0.29	-0.63 ; 0.04	0.082	0.09	-0.22 ; 0.41	0.549	-0.20	-0.52 ; 0.12	0.218
Mean agreement of the regional cardiologists with statement 1	-0.16	-0.79 ; 0.47	0.620						
Mean agreement of the regional cardiologists with statement 2				-0.04	-0.68 ; 0.59	0.889			
Mean agreement of the regional cardiologists with statement 3							-0.22	-0.58 ; 0.15	0.241
Intercept	2.48	-0.62 ; 5.59	0.114	1.74	-1.23 ; 4.71	0.247	3.13	0.86 ; 5.39	0.007**
*Random Effects (region)*									
σ²	1.80			1.45			1.69		
τ_00_	0.00			0.34			0.00		
ICC	0.00			0.19			NA		
N (regions)	15			15			15		
Observations	108			107			109		
Marginal R²/Conditional R²	0.099/0.099			0.225/0.372			0.075/NA		

* p < 0.05 | ** p < 0.01 | *** p < 0.001

The explanatory power of the regression models was mixed. The conditional R² was 0.37 for statement 2, but lower for statement 1 (0.099) and unavailable for statement 3 due to singularity of random effects. The response to statement 1 was influenced by age, with older respondents agreeing slightly more with statement 1 (Estimate (b) = 0.04, 95%-CI [0.01–0.07], p = 0.01). No differences between geographical regions were found (ICC = 0.00, Marginal R² = 0.099, Conditional R² = 0.099).

The response to statement 2 was significantly influenced by age, the state and the individual respondent’s score on the self-perceived opinion leadership scale: Older respondents agreed slightly more with the statement (Estimate (b) = 0.03, 95%-CI [0.00; 0.06], p = 0.03). Respondents in the state of Baden-Wuerttemberg agreed less with the statement than those from Rhineland-Palatinate or Saarland (Estimate (b) = -1.18, 95%-CI [-1.78; -0.58], p < 0.001). Respondents who had a higher score on the scale on self-perceived opinion leadership also tended to agree more with statement 2 (Estimate (b) = 0.29, 95%-CI [0.01–0.06], p = 0.04). The random effects section showed a slight influence of the region a physician was based in with an ICC of 0.19, a Marginal R² of 0.23 and a Conditional R² of 0.37.

The response to statement 3 was not related to any of the examined predictors. Since the data indicated singularity of random effects, no regional effects could be calculated either (Marginal R² = 0.08).

## Discussion

A majority of GPs in our study mentioned specific cardiovascular opinion leaders and in most cases these opinion leaders were cardiologists outside the GP’s practice. Similar findings were reported in other research in general practice [26]. Our results suggest that GPs tend to seek an outside opinion on cardiovascular care and do not necessarily take the lead in this field. This backs up research by Grimshaw et al. (2006), who used an instrument on self-designated opinion leadership and found that only about 20% of participating GPs were clearly self-designated opinion leaders [27].

Still, opinion leaders on cardiovascular topics were not identified by all participants in general practices: more than 30% of our respondents did not mention any opinion leader. This is consistent with other research, which showed that opinion leaders are not always identified and that the role of opinion leaders may rotate between individuals [2]. Likewise, research in surgery indicated that opinion leaders change over time [28].

The most striking finding may be that opinion leadership and opinion seeking behaviours were relatively independent from each other, suggesting that these are independent constructs. The authors of the opinion leadership and opinion seeking scales pointed out that opinion leadership and opinion seeking are not mutually exclusive or highly correlated concepts [19]. They argued that opinion leaders also need to gather information on the respective topic. Our study confirms this for medical doctors: A GP might seek a medical specialist’s opinion and then, in return, could be perceived as opinion leading by their staff or other practices due to the knowledge gained from the respective specialist. As highlighted by Flodgren et al. [3] as well as Rogers [29], such influence is not solely dependent on the individual’s (formal) position in an organization, but on their (de-facto) competence and expertise in a given field. Hence, even though we may not assume that a GP will eventually influence specific medical views of a medical specialist, this is not simply related to a formal or hierarchical position, but to a higher grade of specialization in and knowledge of the respective field. In return, for example, a GP might influence the opinion of a medical specialist when it comes to a more holistic assessment of a patient, since the GP typically can be assumed to have more and thematically broader contacts with the patient than the specialist does.

Both GPs and cardiologists tended to agree with the three statements derived from cardiology guidelines. Significant differences were present only when it came to the prescription of highly-dosed statins for Coronary Heart Disease (CHD)-patients. In Germany, GPs and cardiologists hold different views on the use of statins, with GPs being perceived as more reluctant to prescribe statins: For example, a study in 2019 showed a slight tendency that patients had lower odds of receiving a statin after acute myocardial infarction when the prescribing physician was a primary care physician [30].

While our results showed no differences between GPs and cardiologists regarding the other statements, GPs’ agreement still was not significantly predicted by the local cardiologists’ opinion at all. Instead, we found singular effects of age, state, region and self-perceived opinion leadership of the GPs themselves. This might either indicate a coincidental agreement between cardiologists and GPs independent from any opinion leadership issues or it can be interpreted as another indicator for the range of opinion seeking: Even though GPs primarily deemed themselves opinion-seeking and mainly named cardiologists as opinion leaders, they are self-confident physicians who do not make their medical opinions dependent from what the local medical specialists think. Additionally, even though GPs often refer their patients to local medical specialists to avoid additional burden for the patients in terms of traveling, this does not necessarily imply that the GPs view these specific specialists as opinion leaders. What further needs to be considered in this regard is the variety of additional ways physicians can gather information today whenever a complex or controversial issue emerges: Medical guidelines are constantly available online in their current versions and can be accessed and compared at any time, giving physicians the chance to identify any possible dissonances and form their own opinion based on the suggestions by the various guidelines. Additionally, advanced training measures such as quality circles allow for in-depth discussions of specific issues with peers whenever guidance might be needed. Questions can be critically discussed with physicians of the same field and opinions can be formed. Despite the specialization, the opinion of the medical specialist here is not necessarily the more informed one anymore and GPs may not see a strong need to just resort to a medical specialist when it comes to uncertainties. GPs’ self-designation as opinion seekers may thus not play out in distinct opinion-seeking behaviours regarding medical specialists in practice.

Practical relevance of our results especially lies in the independence of opinion leading and opinion seeking we observed: Approaches that aim at disseminating knowledge, new routines or standards among physicians through opinion leaders often implicitly presume that there are certain, established opinion leaders who are followed by other, opinion seeking physicians. We were able to show that these assumptions often do not incorporate that opinion seekers can be leaders as well and that this role is rather dependent on context instead of any of the person’s fixed characteristics. Interventions utilizing opinion leaders must hence consider that just because a given physician has been opinion leading on a given topic in the past, they will not necessarily be leading on other (new) topics as well and that being an opinion leader does not imply that opinion seekers will, in fact, adapt to their views.

## Strengths & limitations

We were able to assess opinion leadership on cardiovascular issues in GPs from various perspectives – our working definition of opinion leadership allowed us to assess opinion leadership in GPs who, by their mere position in the hierarchy compared to medical specialists, might not be considered as potential cardiovascular opinion leaders at all when using other approaches. Self-reported scales from general practitioners, identification of external opinion leaders, comparisons between GPs and cardiologists and possible connections between self-perceived opinion seeking and views on different guideline-derived statements on cardiovascular care allowed for a more in-depth exploration of opinion leadership and seeking compared to approaches that solely rely on, e.g., naming perceived opinion leaders.

One limitation of the study is that the scales we used to calculate self-perceived opinion leadership do not originate in health services research. Even though we saw no explicit aspect that prohibited a transfer to this context and found a high internal consistency, there might be unknown factors that harm the scales’ informative value in a medical environment. The total response rate of 17.50% among GPs and 21.16% among cardiologists is a limitation as well, because it possibly implies selection bias. Furthermore, we cannot rule out that some respondents answered in a socially desirable way and that they were not alone and unaffected by others when filling out the questionnaire.

## Conclusion

When it comes to points of reference regarding cardiovascular issues, the majority of general practitioners identifies opinion leaders – in most cases these are cardiologists. Self-perceived opinion leadership on cardiovascular issues is present with GPs, but opinion seeking prevails nonetheless. These results indicate that even though general practitioners have a different and possibly more in-depth kind of relationship with their patient compared to medical specialists, they still tend to recognize the latter’s more specialized qualifications and seek their opinions. However, in practice this must not be confused with a general or even blind adoption of the specialists’ opinions per se, as opinion seeking and leading are highly dependent on context and opinions can be gathered from various sources. Further research seems valuable especially regarding general practitioners who cannot identify any opinion leaders at all and who do not see themselves as opinion leading either, since it is not clear which points of reference they resort to when it comes to more specific or controversial medical issues. Furthermore, it seems useful to explore where physicians find the persons they deem influential in the first place and whether different settings (e.g. large cities vs. rural areas) lead to different outcomes when it comes to the formation of clinical opinions.

## Electronic supplementary material

Below is the link to the electronic supplementary material.

Supplementary Material 1

## Data Availability

The dataset supporting the conclusions of this article is not publicly available due to ongoing research. However, it is available for research purposes from the corresponding author upon reasonable request.

## List of abbreviations

**BNP**: Brain natriuretic peptide
**CI**: Confidence interval
**GP**: General practitioner
**ICC**: Intraclass correlation coefficient
**IQR**: Interquartile range
**n**: Number of cases
**p**: P-value
**SD**: Standard deviation
**SE**: Standard error
