# Supplementary material for: Opinion seeking behaviour of healthcare providers in ambulatory cardiovascular care in Germany: a cross-sectional study
Source: BMC Health Serv Res. 2022 Nov 23;22:1404. doi: 10.1186/s12913-022-08667-y (PMC9686109; doi:10.1186/s12913-022-08667-y)
Supplement: Supplementary file 1 — Supplementary Material 1 [file 12913_2022_8667_MOESM1_ESM.docx]

**Additional file 1: Questions on opinion leadership and on potentially controversial issues in cardiovascular care as they were presented in the questionnaires (English translation below).**

# Teil 4: Einstellungen zu kardiovaskulären Fragestellungen

|  | Stimme überhaupt nicht zu | |  | | | Stimme voll und ganz zu | |
| --- | --- | --- | --- | --- | --- | --- | --- |
| 4.1 Meine Einschätzung zu kardiovaskulären Fragestellungen scheint für Kollegen unwichtig zu sein. |  |  | |  |  | |  |
| 4.2 Wenn Kollegen Rat zu kardiovaskulären Fragestellungen suchen, fragen sie mich nicht. |  |  | |  |  | |  |
| 4.3 Kollegen kommen häufig zu mir um sich Rat zu kardiovaskulären Fragestellungen zu holen. |  |  | |  |  | |  |
| 4.4 Kollegen treffen Entscheidungen zu kardiovaskulären Fragestellungen basierend auf meinen Ratschlägen. |  |  | |  |  | |  |
| 4.5 Ich überzeuge häufig Kollegen, bei kardiovaskulären Fragestellungen meinem Vorgehen zu folgen. |  |  | |  |  | |  |
| 4.6 Ich beeinflusse häufig die Sicht von Kollegen auf kardiovaskuläre Fragestellungen. |  |  | |  |  | |  |
| 4.7 Bei kardiovaskulären Fragestellungen frage ich Kollegen um Rat. |  |  | |  |  | |  |
| 4.8 Ich brauche keinen Austausch mit Kollegen um Entscheidungen zu kardiovaskulären Fragestellungen zu treffen. |  |  | |  |  | |  |
| 4.9 Ich frage selten Kollegen zum Vorgehen bei kardiovaskulären Fragestellungen. |  |  | |  |  | |  |

|  | Stimme überhaupt nicht zu | |  | | | Stimme voll und ganz zu | |
| --- | --- | --- | --- | --- | --- | --- | --- |
| 4.10 Ich hole gerne die Meinung von Kollegen ein bevor ich Entscheidungen bei kardiovaskulären Fragestellungen treffe. |  |  | |  |  | |  |
| 4.11 Ich fühle mich wohler, Entscheidungen bei kardiovaskulären Fragestellungen zu treffen, wenn ich vorher die Meinungen von Kollegen dazu gehört habe. |  |  | |  |  | |  |
| 4.12 Wenn ich eine Entscheidung bei kardiovaskulären Fragestellungen treffe, sind mir die Meinungen von Kollegen nicht wichtig. |  |  | |  |  | |  |
| 4.13 Gibt es aktuell eine Fachperson, die Ihre Meinung zu kardiovaskulären Erkrankungen prägt? | Ja, einen Arzt innerhalb der Praxis  Ja, einen Hausarzt außerhalb der Praxis  Ja, einen Kardiologen außerhalb der Praxis  Ja, jemand anderen außerhalb der Praxis  Nein, eine solche Person kann ich nicht benennen | | | | | | |

Bitte kreuzen Sie nachfolgend an, wie stark Sie spontan den jeweiligen Aussagen zu kardiovaskulären Themen zustimmen.

|  | Stimme überhaupt nicht zu | |  | | | Stimme voll und ganz zu | | Ich habe keine Meinung dazu |
| --- | --- | --- | --- | --- | --- | --- | --- | --- |
| 4.14 Bei KHK-Patienten ist eine Statintherapie in hohen Dosen einzuleiten. |  |  | |  |  | |  |  |
| 4.15 Bei Patienten mit Atemnot und Verdacht auf Herzinsuffizienz in der Hausarztpraxis ist immer ein BNP-Wert zu bestimmen. |  |  | |  |  | |  |  |
| 4.16 Jeder Patient mit KHK und Hypertonie sollte einen Zielwert von systolisch kleiner 130 mmHG erreichen. |  |  | |  |  | |  |  |

**English version (please note that this translation has only been manufactured to allow international readers to understand the questions – the questions used in the study were in German and are presented above):**

# Part 4: Attitudes towards cardiovascular issues

|  | Do not agree at all | |  | | | Fully agree | |
| --- | --- | --- | --- | --- | --- | --- | --- |
| 4.1 My opinion on cardiovascular issues seems to be unimportant for colleagues. |  |  | |  |  | |  |
| 4.2 Whenever colleagues look for advice on cardiovascular issues, they do not ask me. |  |  | |  |  | |  |
| 4.3 Colleagues often turn to me for advice on cardiovascular issues. |  |  | |  |  | |  |
| 4.4 Colleagues decide on cardiovascular issues based on my advice. |  |  | |  |  | |  |
| 4.5 When it comes to cardiovascular issues, I often convince colleagues to follow my approach. |  |  | |  |  | |  |
| 4.6 I often influence the views of colleagues on cardiovascular issues. |  |  | |  |  | |  |
| 4.7 When it comes to cardiovascular issues, I turn to colleagues for advice. |  |  | |  |  | |  |
| 4.8 I do not need an exchange of views with colleagues to decide on cardiovascular issues. |  |  | |  |  | |  |
| 4.9 I rarely ask colleagues about how to approach cardiovascular issues. |  |  | |  |  | |  |

|  | Do not agree at all | |  | | | Fully agree | |
| --- | --- | --- | --- | --- | --- | --- | --- |
| 4.10 I like to hear colleagues’ opinions before I decide on cardiovascular issues. |  |  | |  |  | |  |
| 4.11 I feel more comfortable with deciding on cardiovascular issues when I heard colleagues’ opinions beforehand. |  |  | |  |  | |  |
| 4.12 When I decide on cardiovascular issues, the opinions of colleagues are not important to me. |  |  | |  |  | |  |
| 4.13 Is there currently a professional who affects your opinion on cardiovascular conditions? | Yes, a physician within my practice  Yes, a GP outside of my practice  Yes, a cardiologist outside of my practice  Yes, someone else outside of my practice  No, I can’t name such a person | | | | | | |

Please indicate how much you spontaneously agree with the following statements on cardiovascular topics.

|  | Do not agree at all | |  | | | Fully agree | | I don’t have an opinion on that |
| --- | --- | --- | --- | --- | --- | --- | --- | --- |
| 4.14 For patients suffering from coronary heart disease, a therapy with highly dosed statins has to be initiated. |  |  | |  |  | |  |  |
| 4.15 The general practice always has to check the BNP-parameter when a patient suffers from dyspnoea and possibly suffers from heart failure. |  |  | |  |  | |  |  |
| 4.16 Every patient with coronary heart disease and hyper-tension needs to reach a systolic reading below 130 mmHg. |  |  | |  |  | |  |  |
